# Differential expression of miRNAs and their target genes in senescing leaves and siliques: insights from deep sequencing of small RNAs and cleaved target RNAs

**DOI:** 10.1111/pce.12393

**Published:** 2014-08-13

**Authors:** SHAWN R THATCHER, SHAUL BURD, CHRISTOPHER WRIGHT, AMNON LERS, PAMELA J GREEN

**Affiliations:** 1 Delaware Biotechnology Institute, University of DelawareNewark, DE, 19711, USA; 2 Department of Postharvest Science of Fresh Produce, Agricultural Research Organization, Volcani CenterBet Dagan, 50250, Israel

**Keywords:** *Arabidopsis thaliana*, microRNA, nutrient remobilization, PARE, senescence, silique

## Abstract

MicroRNAs (miRNAs) are a class of small RNAs, which typically function by guiding cleavage of target mRNAs. They are known to play roles in a variety of plant processes including development, responses to environmental stresses and senescence. To identify senescence regulation of miRNAs in *Arabidopsis thaliana*, eight small RNA libraries were constructed and sequenced at four different stages of development and senescence from both leaves and siliques, resulting in more than 200 million genome-matched sequences. Parallel analysis of RNA ends libraries, which enable the large-scale examination of miRNA-guided cleavage products, were constructed and sequenced, resulting in over 750 million genome-matched sequences. These large datasets led to the identification a new senescence-inducible small RNA locus, as well as new regulation of known miRNAs and their target genes during senescence, many of which have established roles in nutrient responsiveness and cell structural integrity. In keeping with remobilization of nutrients thought to occur during senescence, many miRNAs and targets had opposite expression pattern changes between leaf and silique tissues during the progression of senescence. Taken together, these findings highlight the integral role that miRNAs may play in the remobilization of resources and alteration of cellular structure that is known to occur in senescence.

## Introduction

In plants, senescence is a broad term that describes whole plant as well as tissue-specific changes that take place over the course of aging. During senescence, the leaf's cellular structure, metabolic activities and physiological role are greatly altered. Chloroplasts degenerate and the photosynthetic apparatus disassembles (Hörtensteiner 2006; Thomas 2013). Senescence is also characterized by widespread and significant changes in the pattern of gene expression (Wagstaff *et al*. 2009; Breeze *et al*. 2011). The expression of many genes, such as those associated with photosynthesis, is repressed, while that of many other genes, termed senescence-associated genes (SAGs), is induced. The molecular mechanisms governing senescence regulation have been suggested to form a complex network responsible for activation of the different SAGs (Guo & Gan 2005). Various SAGs exhibit differential expression in different tissues and in response to different senescence-promoting stimuli, including hormones and abiotic stress (Park *et al*. 1998; Weaver *et al*. 1998; Lim *et al*. 2007; Fischer 2012). Senescence regulation involves the action of a large number of transcription factors and epigenetic programming via histone modifications (Gregersen & Holm 2007; Balazadeh *et al*. 2008; Ay *et al*. 2009; Breeze *et al*. 2011; Brusslan *et al*. 2012). Much less is known about post-transcriptional control during senescence.

Because of the tight regulatory control exerted during the progression of senescence, it can be seen as a final developmental stage (Gan & Amasino 1997). In annual plants, including *Arabidopsis*, leaf senescence is thought to be a response to dwindling nutrient availability as each growing season comes to a close. Faced with a limited supply of micro and macronutrients, plants direct senescence in leaves in order to mobilize resources, such as copper, phosphate and nitrate, into reproductive tissue to enhance seed yield (Himelblau & Amasino 2001; Hörtensteiner & Feller 2002; Diaz *et al*. 2008; Gregersen *et al*. 2008). This idea is known as the nutrient relocalization hypothesis (Gan & Amasino 1997; Guiboileau *et al*. 2010). Given their established role in responding to nutrient availability (Chiou *et al*. 2006; Abdel-Ghany & Pilon 2008; Kant *et al*. 2011), microRNAs (miRNAs) could easily play a crucial and currently underappreciated part in guiding this process.

miRNAs are a class of small RNAs (sRNAs) that typically function by guiding ARGONAUTE-mediated cleavage of target mRNAs. They have been shown to regulate a host of processes ranging from development to responses to nutrient levels, such as phosphate and nitrate (Chiou *et al*. 2006; Kant *et al*. 2011). miRNAs were directly implicated in regulating *Arabidopsis* senescence when it was shown that miR164 is a key player in the senescence regulatory pathway. Natural reduction of miR164 level during senescence results in increased cell death because of its control on its target, the senescence transcriptional activator gene *ORE1* (Kim *et al*. 2009). However, no large-scale sequencing of sRNAs or analysis of their target genes over the course of senescence has been presented thus far.

In addition to changes in transcript abundance, senescence is also thought to result in broad changes in RNA decay through increased expression of ribonucleases (RNases; Taylor *et al*. 1993; Bariola *et al*. 1999). It has been proposed that this increase in RNA turnover may be an adaptive response to senescence-related changes in phosphate levels (Bariola *et al*. 1994; Green 1994). Construction and sequencing of parallel analysis of RNA ends (PARE) libraries enables the precise examination of decay intermediates generated by miRNA-guided cleavage events (German *et al*. 2008). When a miRNA guides an AGO protein to cleave its target mRNA, the downstream cleavage product has a monophosphorylated 5′ end which PARE is designed to capture. Thus, through the analysis of PARE data, a clearer picture of the RNA degradome during the course of senescence can be obtained.

In this study, we utilize high-throughput sequencing of sRNA libraries constructed from both senescing leaf and silique tissue to discover differential miRNA expression in *Arabidopsis*. Expression levels of target genes for many regulated miRNAs were also examined in detail, and often found to exhibit tissue-specific regulation as senescence progressed. This analysis was complemented by the construction and sequencing of PARE libraries, which enabled examination of miRNA-guided cleavage events during senescence. The combination of sRNA, target mRNA and PARE data led to a much more complete picture of the roles that miRNAs may play in *Arabidopsis* senescence.

## Materials and Methods

### Plant growth and stress treatments

**Figure 1:**
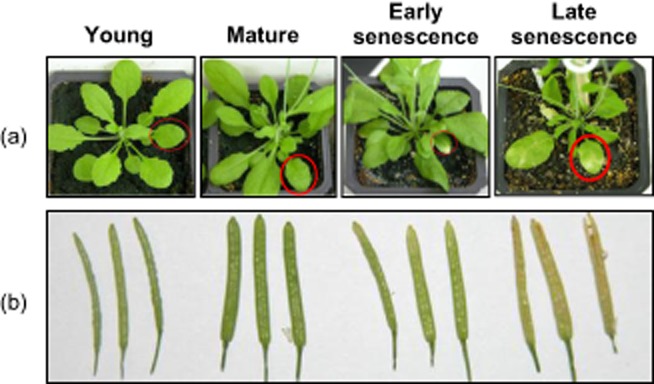
Leaves and siliques over the course of development and senescence. The course of development and senescence is represented by four stages. (a) Leaves at four stages: young (35 d), mature (45 d), early senescence (50 d), late senescence (55 d). (b) Siliques at four stages determined and selected based on visual inspection as described in methods.

Plant material was harvested from *Arabidopsis* Col-0 wild-type plants. Plants were grown in short days (8 h) for 2 weeks on 1/2 × MS 0.8% agar plates. Seedlings were transferred to artificial soil in 7 × 7 × 8 cm containers and grown with a light/dark cycle of 16/8 h (long days). For leaf tissue, leaves five and six were harvested from multiple plants and pooled at four different stages: young (20 d after transfer), mature (30 d after transfer), early senescence (35 d after transfer) and late senescence (40 d after transfer). Silique tissue was also harvested from multiple plants and pooled. It was staged based on visual morphology indicative of senescence and developmental stage, including increased diameter, progression of chlorotic tissue and splitting (Fig. 1a). All tissues were immediately frozen in liquid nitrogen and stored at −80 °C. RNA was extracted from leaves or siliques using Trizol reagent (siliques, Molecular Research Center, Cincinnati, Ohio, USA) or Plant RNA Isolation Reagent (leaves, Invitrogen, Carlsbad, CA, USA), according to the manufacturers' protocols.

### sRNA library construction and sequencing

sRNA libraries were constructed as described previously with minor modifications (Lu *et al*. 2007). Briefly, sRNA was isolated from total RNAs using PEG8000/NaCl-mediated low molecular weight RNA precipitation and then run on a 15% denaturing PAGE gel to select and extract 20–30 nt RNAs. 5′ and 3′ adaptors were ligated, followed by additional PAGE purification after each reaction. RNA-containing adaptors were reverse transcribed and amplified via 18 cycles of PCR followed by PAGE purification to select the final product. RNA and DNA oligos used to prepare the libraries are listed in Supporting Information Table S1. Libraries were sequenced using an Illumina IIGX platform, Illumina, San Diego, CA, USA, with either one sample per lane (leaf sequencing data) or two barcoded samples per lane (silique sequencing data).

### Analysis of sRNA sequencing data

Sequencing data from sRNA libraries were trimmed to remove adaptor sequences using a custom perl script, and then mapped to the *Arabidopsis* genome [The Arabidopsis Information Resource (TAIR) version 10; http://www.arabidopsis.org] with Bowtie, with no mismatches allowed (option -v 0). Reads matching perfectly to the genome and not matching tRNA, rRNA and snoRNA (as annotated in TAIR10) were retained for further analysis. These reads were normalized to transcripts per two million (TP2M) to account for varying library sequencing depth. Reads were then compared with known miRNAs from miRBase version 19 (http://www.mirbase.org) and only perfect matches to annotated miRNAs were used for quantification. Remaining sRNAs, which did not match to known miRNAs and were elevated threefold or more in early or late senescence leaf libraries compared with mature leaf libraries, were considered as possible new senescence-inducible sRNAs. These were further examined as described in the text and the RNA blot section below.

### PARE RNA library construction, sequencing and processing

PARE libraries were constructed as previously described with minor modifications (German *et al*. 2008, 2009). Briefly, 300 *μ*g of total RNA was poly-A selected using oligo d(T)_25_ magnetic beads (New England BioLabs, Inc., Beverly, MA, USA). A 5′ RNA adaptor (Supporting Information Table S1) was ligated onto the remaining RNA, which was subjected to a second poly-A selection. The ligated RNA was then reverse transcribed using an oligo d(T)_18_ primer (New England BioLabs, Inc.) and phenol-chloroform extracted to select for cDNA. The cDNA was subjected to a short (8-cycle) PCR amplification before being digested with MmeI (New England BioLabs, Inc.), resulting in a 42 nt fragment containing 5′ adaptor and 20 nt of the original poly-A selected mRNAs. An RNA duplex complimentary to the 5′ adaptor sequence (Supporting Information Table S1) was then ligated on and the resulting product was amplified by 20 cycles of PCR and size selected on a 6% PAGE gel. After purification from the gel, the final library product was sequenced using an Illumina IIGX platform. Sequencing data from PARE libraries were trimmed to remove adaptors using the same custom perl script that was used in sRNA trimming and then mapped to the *Arabidopsis* genome using Bowtie, with no mismatches allowed (option -v 0; TAIR version 10; http://www.arabidopsis.org). Reads matching perfectly to the genome were used for further analysis.

### Prediction and validation of targets

Targets were predicted for the abundant sRNAs arising from the senescence-inducible sRNA *(sen-sRNA)* locus using psRNATarget (Dai & Zhao 2011). Targets with scores <4.0 were considered potential new targets. To validate predicted cleavage, senescence PARE sequencing data as well as PARE sequencing data from *xrn4* flowers obtained from NCBI's Gene Expression Omnibus with accession number GSM280227 were utilized. Sequences matching to cleavage products starting between bases 10 and 11 from the 5′ end of the predicted sRNA pairings were considered as evidence for sRNA-guided cleavage and were obtained through use of a custom perl script.

### RNA blot analysis

miRNAs and other sRNAs with potential expression changes in sRNA sequencing data were subjected to northern blotting in at least two biological replicates. One replicate corresponded to the same RNA used for sRNA sequencing in the majority of cases. sRNA analyses were carried out by running low molecular weight RNA on a 15% denaturing PAGE gel. RNA was transferred onto Hybond N + membrane (Amersham, Piscataway, NJ, USA) and fixed using ultraviolet (UV) cross linking. Membranes were then pre-hybridized for at least 2 h in Ultrahyb-Oligo hybridization buffer (Ambion, Austin, TX, USA). DNA oligos complementary to the miRNA of interest were labelled with [у32P]ATP using Optikinase (Amersham Biosciences), filtered using QIAquick nucleotide removal kit (Qiagen, Valencia, CA, USA). Labelled probes were added to the hybridization solution and allowed to hybridize for at least 12 h. mRNA blots were carried out by running 20 *μ*g of total RNA onto 1.5% formaldehyde agarose gels, followed by transfer onto Hybond N^+^ membranes. Membranes were then pre-hybridized overnight using hybridization buffer (Church & Gilbert 1984) and probed with ^32^P labelled probes. The probes were generated by PCR using primers described in Supporting Information Table S1. mRNAs were visualized using a Typhoon phosphorimager (GE Healthcare Life Sciences, Piscataway, NJ, USA).

### Splinted-ligation-mediated miRNA detection

miRNAs were examined by miRtect-IT miRNA labelling and detection kit (Affymetrix, Santa Clara, CA, USA) as described previously (Maroney *et al*. 2008; Jeong & Green 2012). In brief, miRNAs were captured from 2 *μ*g of total RNA using a bridge oligonucleotide that is designed to specifically detect one specific miRNA and ligated to a ^32^P labelled detection oligo using T4 DNA ligase. The resulting products were separated via a 15% PAGE gel and visualized using a Typhoon phosphorimager (GE Healthcare Life Sciences).

### GEO accession

sRNA and PARE data have been added to the National Center for Biotechnology Information Gene Expression Omnibus (NCBI GEO) (GSE55151).

## Results

### sRNA libraries from senescing Arabidopsis leaves and siliques

In order to examine the expression patterns of miRNAs during *Arabidopsis* senescence, sRNA libraries were constructed from leaves and siliques at four different developmental stages. Leaves five and six were selected based on age after germination (Fig. 1a): young leaves (35 d), mature leaves (45 d), early senescing leaves (50 d) and late senescing leaves (55 d). Siliques were selected based on visual morphology indicative of developmental and senescence stage, including diameter, chlorosis and splitting (Fig. 1b). After trimming of adaptors and removal of rRNA, tRNA and snoRNAs, these eight sRNA libraries yielded more than 200 million genome-matched sequences (Table 1), and represent the first massive sRNA sequencing from senescing plant tissues. After normalization to transcripts per two million (TP2M), the total abundance of sRNAs in each size class were plotted. In leaf, 21 nt sRNAs predominated, followed by 24 nt RNAs, while in silique, 24 nt sRNAs predominated, followed by 21 nt (Supporting Information Fig. S1). This distribution difference stems from leaf tissue possessing relatively more miRNAs, which are mostly 21 nt, compared with 24 nt siRNAs. The average total miRNA abundance for leaf libraries was over 1 000 000 TP2M, while in silique, it was only about 83 000 TP2M, indicating that miRNAs are the major type of sRNA in leaf but not silique tissues (Supporting Information Table S2).

**Table 1:** Summary statistics of small RNA libraries

Library	Tissue	Description	Raw trimmed	Genome matched		
			Reads	Reads	Distinct	t/r/sn/snoRNA
C0LY	Leaf	Young	27 870 710	17 840 547	1 021 881	6 783 580
C0LM	Leaf	Mature	26 211 889	16 604 542	473 673	7 452 607
C0LSeE	Leaf	Early senescence	28 700 595	17 875 180	212 951	6 434 947
C0LSeL	Leaf	Late senescence	29 146 047	11 853 598	97 227	12 456 070
C0SY	Silique	Young	33 654 351	30 804 353	4 071 246	8 612 996
C0SM	Silique	Mature	43 310 733	38 683 184	6 700 592	7 112 309
C0SSeE	Silique	Early senescence	22 880 129	20 049 781	3 957 517	3 251 398
C0SSeL	Silique	Late senescence	74 829 453	64 627 925	8 320 789	13 003 515

Genome-matched abundance and distinct represent sequences, which remained after removal of t/r/sn/snoRNAs.

### Differential expression of known miRNAs during senescence

Normalized miRNA expression levels from young, early senescence and late senescence leaf and silique libraries were then compared with expression in the respective mature tissue to determine if any miRNAs were differentially expressed (Supporting Information Table S2). As an initial screen for regulated candidates, the abundance of annotated miRNAs in mature leaf and silique libraries with abundance greater than 50 transcripts per two million (TP2M) in any library were plotted against their abundance in young, early senescence or late senescence libraries (Supporting Information Fig. S2). In general, the expression of miRNAs across libraries was highly similar, with mature versus young showing the strongest miRNA expression correlation in both leaf and silique libraries (*R*^2^ of 0.95 and 0.94, respectively) and in mature versus late senescence leaf and silique libraries showing the weakest correlation (*R*^2^ of 0.79 and 0.81, respectively). Despite mostly similar expression patterns, certain miRNAs did show marked increases or decreases in early and late senescing tissue (Supporting Information Table S2).

**Figure 2:**
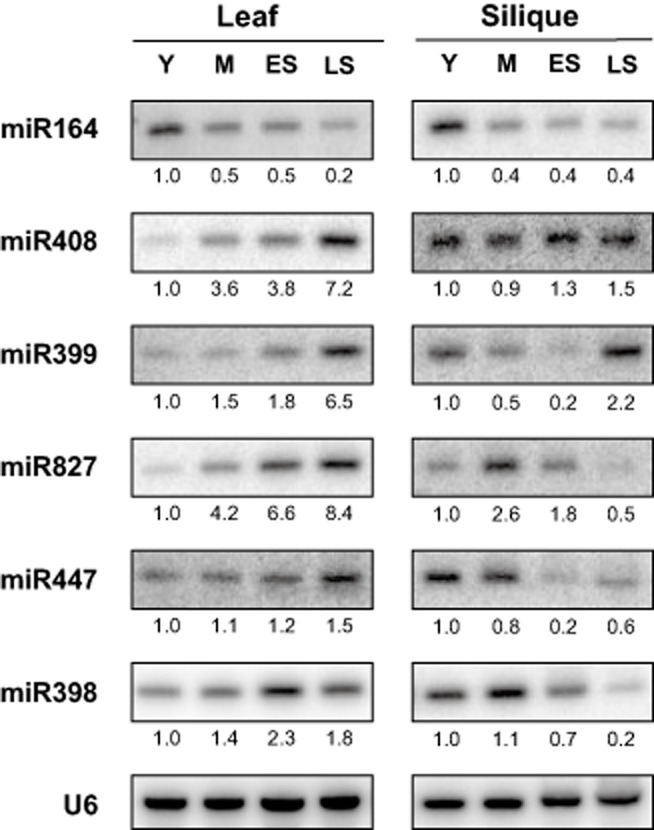
Expression of nutrient-responsive miRNAs in Arabidopsis leaf and silique over the course of senescence and development. miRNA expression level was examined via northern blotting. Stages Y (young), M (mature), ES (early senescence) and LS (late senescence) were selected as described in Fig. 1. U6-normalized quantification values are displayed below each stage. Data are representative of at least two biological replicates; one representative U6 is shown.

miRNAs showing expression changes greater than threefold from mature to early or late senescence were confirmed in two or more independent biological replicates via northern blotting or splinted ligation-mediated detection (Jeong & Green 2012). miR164, which targets the positive regulator of senescence-induced cell death *ORE1* (*AT5G39610*), has been previously reported to decline in leaves as senescence progresses (Kim *et al*. 2009). Both sRNA sequencing data and sRNA northern blot validation reproduced this regulation, showing a steady decline from young to late senescence stages in leaves (Fig. 2). Interestingly, miR164 was reduced from young to mature siliques but did not continue to decline as senescence progressed in this tissue (Fig. 2) indicating that levels of senescence-responsive miRNAs may change in a tissue-dependent manner.

In addition to reproducing regulation of miR164, differential expression of seven other *Arabidopsis* miRNAs during the course of senescence was observed (Fig. 2). Interestingly, five out of these seven miRNAs have been reported to be nutrient responsive. miR408 targets a host of genes involved in pollen tube guidance, copper metabolism and cell wall integrity (Sunkar & Zhu 2004) and has been shown to be responsive to low copper levels (Abdel-Ghany & Pilon 2008). Both sRNA sequencing data and northern blot validation showed that this miRNA increased approximately sevenfold during senescence in leaf tissue (Fig. 2). However, the expression pattern of miR408 in siliques differed from leaves, only increasing very slightly (Fig. 2).

Several other nutrient-responsive miRNAs also showed different expression trends between leaf and silique during senescence. Both miR399, which targets the negative repressor of phosphate transport, *PHOSPHATE2* (*PHO2*, *AT2G33770*) and miR827, which targets the nitrogen/phosphate balance regulator *NITROGEN LIMITATION ADAPTATION* (*NLA*, *AT1G02860*), are well known to be strongly induced by low phosphate conditions (Chiou *et al*. 2006; Kant *et al*. 2011). Both of these miRNAs increased as senescence progressed in leaves (Fig. 2) but had different expression patterns in siliques. In both cases, a reduction in expression occurred in early senescing siliques compared with mature. In late senescence, miR827 continued to decline, dropping to nearly undetectable levels. miR399, on the other hand, was strongly increased at the end of senescence.

The miR447 precursor has been demonstrated to increase under both phosphate and nitrate limiting conditions and the mature miRNA targets *2-PHOSPHOGLYCERATE KINASE* (*2PGK*, *AT5G60760*), a gene involved in phytic acid metabolism (Allen *et al*. 2005; Pant *et al*. 2009; Kim & Tai 2011). Our results indicated that the mature miRNA increases slightly in leaves during senescence (Fig. 2). In siliques, however, miR447 is reduced at the outset of senescence and then increased in late senescence (Fig. 2). miR398 has been reported to respond to a host of nutrients including phosphate, nitrate and sucrose and targets genes involved in glucose metabolism and the response to oxidative stress (Sunkar *et al*. 2006; Dugas & Bartel 2008; Pant *et al*. 2009). miR398 showed a mild induction as senescence progressed in leaves and a much larger reduction in siliques (Fig. 2).

**Figure 3:**
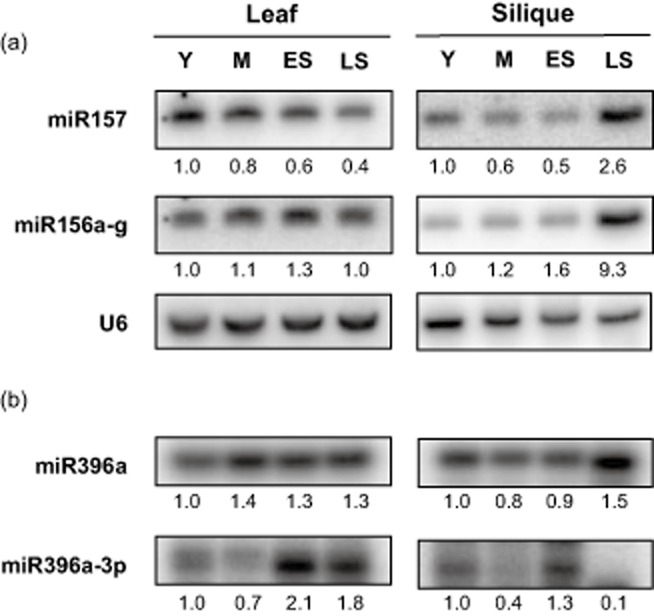
Expression of miRNAs in Arabidopsis leaf and silique. Stage selection and abbreviation as described in Fig. 1. (a) miR156/157 family members' expression level was examined via northern blotting. Data is representative of at least two biological replicates with U6-normalized quantification values are displayed below each stage. (b) miR396a and miR396a-3p expression was examined via splint-ligation-mediated miRNA detection. Quantification numbers are displayed below.

Two other regulated miRNA families without clear roles in nutrient responsiveness also showed senescence regulation (Fig. 3). The miR156/157 family is a highly conserved miRNA family, which targets *SQUAMOSA PROMOTER LIKE BINDING* (*SPL*) genes that are crucial for development (Gandikota *et al*. 2007). Intriguingly, miR157 showed a slight decrease during senescence in leaves while its close relative, miR156 was unchanged (Fig. 3a). Both miR156 and miR157 had different expression patterns in siliques compared with leaves, where they both increased strongly in late senescence. miR396a, which increased very slightly in both leaf and silique senescence (Fig. 3b), was recently found to produce an abundant miRNA*, miR396a-3p in certain tissues (Jeong *et al*. 2013). miR396a-3p had a larger increase in expression in leaves and a steep decline in siliques during late senescence (Fig. 3b), indicating that this newly annotated miRNA may play a previously unknown role during senescence.

### New senescence-inducible sRNA locus targets the alpha tubulin family

**Figure 4:**
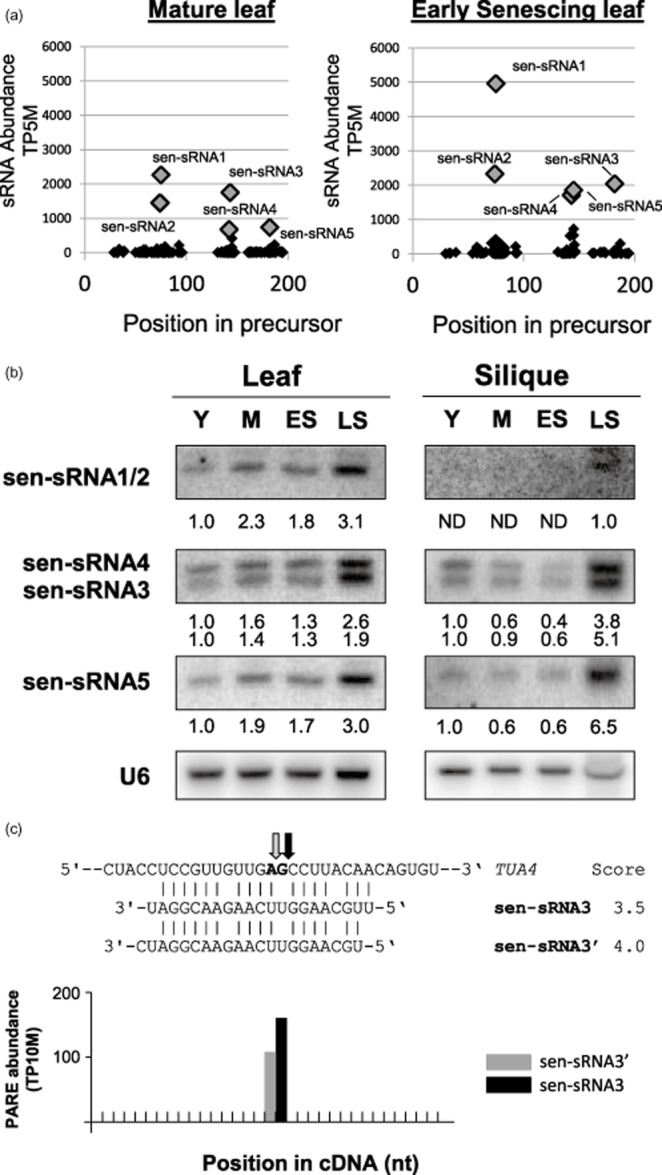
Expression and target cleavage activity of the *sen-sRNA* locus. (a) Small RNA plot of the *sen-sRNA* precursor. The abundance of small RNA sequences is plotted versus position within the predicted precursor of the *sen-sRNA* locus for both mature and early senescing leaf. (b) Expression of sRNAs from the *sen-sRNA* locus were examined via northern blotting. Data is representative of at least two biological replicates and U6-normalized quantification values are displayed below each stage. Stages were as described in Fig. 1. (c) Predicted cleavage sites of sen-sRNA3 and sen-sRNA3′ are indicated by arrows and bold 5′ nucleotides on target mRNAs. Target scores for each sRNA are also shown. Target cleavage data were obtained from PARE data of *xrn4* flower (German *et al*. 2008). PARE abundance is shown in histograms. Grey bars indicates cleavage guided by sen-sRNA3′ while black bars indicate cleavage guided by sen-sRNA3.

Given that no large-scale sequencing of sRNAs has been carried out in senescencing tissue, an analysis of senescence libraries could reveal sRNAs, which are only significantly expressed at this stage. To find new senescence-induced sRNAs, sRNA data were first filtered to remove tRNA, rRNA, snoRNA and previously annotated miRNAs. Regions surrounding the remaining sRNAs, which were elevated threefold or more in senescing leaf libraries compared with mature, were required to have a strand bias of greater than 90%. Remaining regions were then subjected to manual folding analysis using Centroidfold (Sato *et al*. 2009) to determine their ability to form stem-loop structures. After application of these filters, one new sRNA locus was discovered, and named *sen-sRNA* locus (Fig. 4a, Table 2). *sen-sRNA* precursor forms a nearly perfect inverted repeat and generates five abundant sRNAs, sen-sRNA1-5, which were confirmed to be senescence-regulated by northern blotting. All of these sRNAs from the *sen-sRNA* locus increased to their highest level in late senescing leaves (Fig. 4b). In siliques, these abundant sRNAs from the *sen-sRNA* precursor also showed their highest expression levels in late senescence (Fig. 4b).

**Table 2:** Abundant small RNAs from the *sen-sRNA* precursor

miRNA	Sequence	Size	Abundance (TP10M)
sen-sRNA1	UUCUUGAACCUUGGAAGAAAA	21	8361
sen-sRNA2	UCUUGAACCUUGGAAGAAAAC	21	5840
sen-sRNA3	UGCAAGGUUCAAGAACGGAUC	21	5435
sen-sRNA4	UCUUGCAAGGUUCAAGAACGGAUC	24	2347
sen-sRNA5	AUUCGACAAAGUGAAGGGUUU	21	2520

Sequence, size and sum of abundance across all eight small RNA libraries are shown.

Targets were predicted for all the abundant sRNAs arising from the *sen-sRNA* locus using psiRNA target prediction software (Dai & Zhao 2011). Published PARE data from *xrn4* flowers were examined to search for evidence of cleavage for each potential target (German *et al*. 2008). The loss of the exoribonuclease XRN4 in this mutant background causes elevated levels of sRNA-guided cleavage intermediates to accumulate, enabling sRNA targets to be more easily identified (Souret *et al*., 2004; Rymarquis *et al*., 2011; Nagarajan *et al*., 2013). sen-sRNA3 was predicted to target a conserved region of the alpha tubulin family, pairing to *TUA2*, *TUA4* and *TUA6* with scores of 4.0, 3.5 and 4.0, respectively (Fig. 4c). Abundant cleavage products predicted for sen-sRNA3 and a variant that differs by one nucleotide were both found in this library. These decay intermediates could arise from cleavage of one, two or all three alpha tubulin family members, which cannot be distinguished because of the conserved nature of this region.

### Analysis of PARE data from senescing leaves

In addition to utilizing sRNA and publicly available PARE libraries, six new PARE libraries were constructed from *Arabidopsis* leaves to examine targets of regulated miRNAs. After trimming of adaptors, these libraries yielded more than 750 million genome-matched sequences and represent the first large-scale analysis of the RNA degradome during *Arabidopsis* senescence (Table 3). Early and late senescing leaf libraries had significantly reduced genome-matched distinct sequences, despite a second round of library construction and sequencing from biological replicates (Table 3). This reproducible overall reduction in complexity is likely caused by the general increase in RNA decay associated with senescence progression (Taylor *et al*. 1993; Bariola *et al*. 1999). In keeping with this reduction in distinct sequences, the majority of miRNA target cleavage products were either missing or significantly reduced.

**Table 3:** Summary statistics of PARE libraries

Library	Tissue	Description	Raw trimmed	Genome matched	
			Reads	Reads	Distinct
C0LY	Leaf	Young	114 373 290	97 727 510	11 087 211
C0LM	Leaf	Mature	107 307 144	92 663 203	11 592 888
C0LSeE1	Leaf	Early senescence 1	380 719 524	335 224 824	1 689 330
C0LSeL1	Leaf	Late senescence 1	142 841 947	107 151 162	163 022
C0LSeE2	Leaf	Early senescence 2	127 367 565	114 143 220	1 192 825
C0LSeL2	Leaf	Late senescence 2	26 170 444	22 820 583	260 527

**Figure 5:**
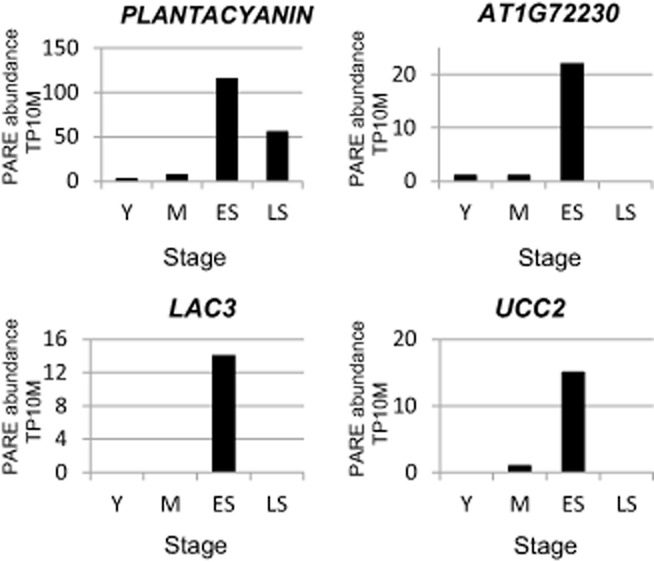
PARE sequences corresponding to precise miR408 target cleavage sites. PARE libraries data showing the level of decay intermediates generated by miR408-guided cleavage of *PLATNACYANIN*, *AT1G72230*, *LAC3* and *UCC2*. Cleavage data were obtained from PARE libraries constructed at four stages during development and senescence. Bars represent the abundance of cleavage products guided by miR408.

Initial analysis of these PARE libraries focused on examining targets of regulated miRNAs. Because of the reduction or absence of most miRNA target cleavage products in senescing leaves, it is not possible to draw conclusions about lower levels of cleavage products for targets whose miRNAs were shown to be reduced as senescence progresses. However, strong induction of miR408 in leaves was accompanied by an increase in the cleavage products generated for all four of its validated targets (Fig. 5). Similarly, NLA had a significant increase in the cleavage product generated by miR827-guided cleavage (data not shown).

### Differential expression of senescence-regulated targets

**Figure 6:**
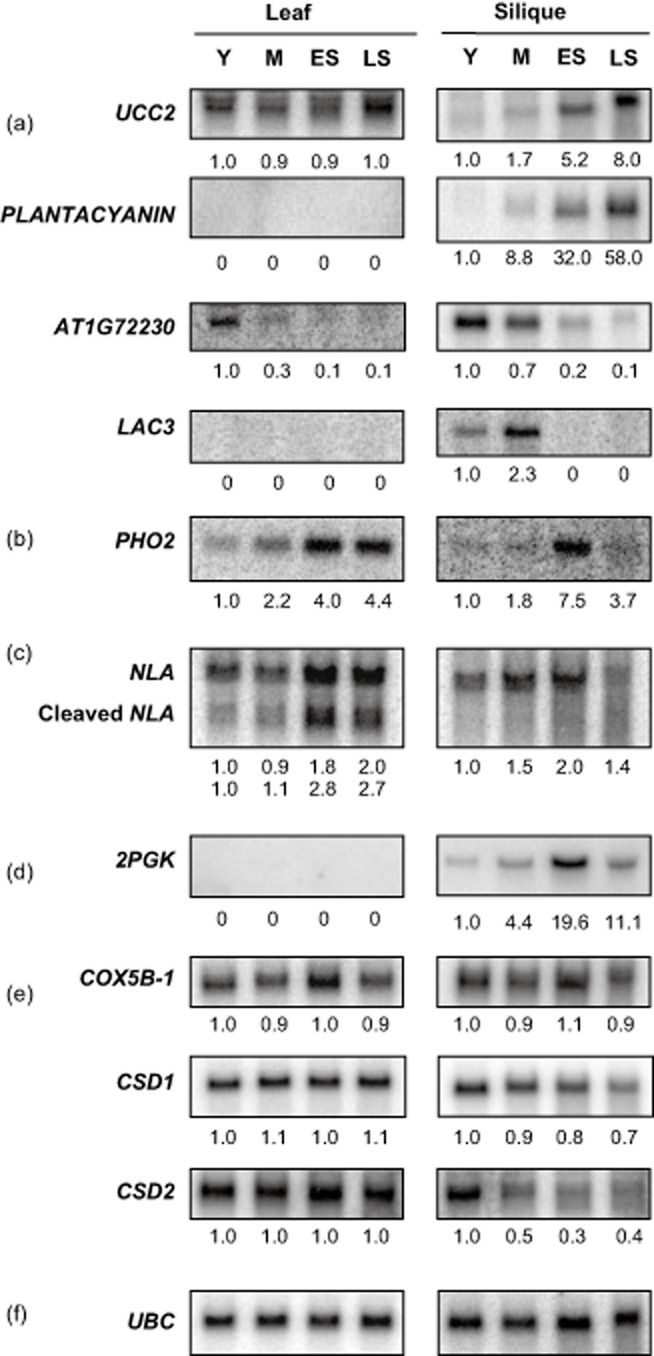
Senescence regulation of nutrient-responsive miRNA targets in leaf and silique. Northern blots showing differential expression of nutrient regulated miRNA targets in senescing leaf and silique. Data is representative of at least two biological replicates. Stages were selected as described in Fig. 1. *UBC* (*At5g25760*) normalized quantification values are displayed below each stage. (a) miR408 targets *UCC2*, *PLANTACYANIN*, *AT1G72230* and *LAC3*; (b) miR399 target *PHO2*; (c) miR827 target *NLA*; (d) miR447 target *2PGK*; (e) miR398 targets *COX5B-1*, *CSD1*; and *CSD2* (f) Representative *UBC*.

Expression changes of the genes targeted by senescence-regulated miRNAs were also analysed. Although the majority of senescence-regulated miRNAs have previously been shown to be nutrient responsive, their targets possessed a wide variety of functions ranging from nutrient mobilization to cell wall integrity. Like many senescence-regulated miRNAs, these targets often showed different expression changes between leaf and silique tissue (Fig. 6). Some of these targets showed incoherent regulation (Jeong & Green 2013) relative to the miRNAs targeting them, that is, they increase despite a simultaneous increase in the miRNAs, which targets them (Figs. 2 & 6).

Four of targets of miR408, which had increased cleavage products in senescence PARE data (Fig. 5), were analysed via northern blotting. *AT1G72230*, a member of the *CUPREDOXIN* superfamily, which binds copper and functions in the synthesis of xylan, a polysaccharide component of cell walls (Borner *et al*. 2002; Mutwil *et al*. 2009), decreased as aging progressed in both leaves and siliques (Fig. 6a). *UCLACYANIN 2* (*UCC2*, *AT2G44790*) also contains a copper-binding domain but its function remains uncertain (Borner *et al*. 2002; Mutwil *et al*. 2009). Unlike *AT1G72230*, *UCC2* was mostly unchanged during leaf senescence and actually increased in senescing siliques (Fig. 6a). *LAC3* (*AT2G30210*) is a member of the laccase family that has been proposed to be involved in maintaining cell wall integrity (Turlapati *et al*. 2011). *LAC3* expression was not detected via northern blotting in leaves, but it was highly expressed in young and mature siliques and decreased rapidly during senescence (Fig. 6a). *PLANTACYANIN* (*AT2G02850*), which is thought to function in pollen tube guiding, has only been reported to be expressed significantly in the pistil and root (Dong *et al*. 2005; Abdel-Ghany & Pilon 2008). Despite a highly abundant cleavage product present in leaf PARE libraries, no expression was detected in this tissue through northern blotting (Fig. 6a). However, *PLANTACYANIN* was detectable in early senescing siliques and increased further during late senescence, indicating incoherent regulation with miR408.

Both miR399, which targets *PHO2*, and miR827, which targets *NLA*, were strongly induced during senescence in leaves (Fig. 2). Interestingly, *PHO2* and *NLA* were themselves increased in senescing leaves, showing incoherent regulation with the miRNAs which target them (Fig. 6b,c). While full-length *NLA* increased during leaf senescence, its putative miR827-guided cleavage product increased even more strongly (Fig. 6c). This result is consistent with an increase of miR827 and an accumulation of miR827 directed decay product in PARE results and implies that miR827 may be functioning to moderate *NLA's* expression increase during senescence instead of acting as an on/off switch. In siliques, *PHO2* and *NLA* showed mostly coherent regulation, increasing to their highest levels in early senescence as miR399 and miR827 decreased.

*2PGK*, a target of miR447 has previously been reported to be flower and silique preferential (Kim & Tai 2011). In keeping with published work, *2PGK* was undetectable in leaves at any point during senescence (Fig. 6d). In siliques, *2PGK* increased from young to early senescence stages, reproducing earlier work demonstrating its role in increased phytic acid levels during silique maturation (Kim & Tai 2011). However, *2PGK* decreased as siliques progressed to late senescence, again showing coherent regulation with miR447 at this stage.

miR398 targets a host of genes involved in a variety of different processes ranging from glucose metabolism to the oxidative stress response. *COX5B-1* (*AT3G15640*), which is part of the glucose catabolic process (Comelli *et al*. 2009), was selected for northern blot analysis based on known macronutrient changes during senescence (Wingler & Roitsch 2008). Surprisingly, the expression of *COX5B-1* was relatively unchanged in leaves or siliques during senescence despite regulation of miR398 itself (Fig. 6e). Other targets of miR398, *SUPEROXIDE DISMUTASE 1*(*CSD1*) and *SUPEROXIDE DISMUTASE 2* (*CSD2*), which are responsible for reducing reactive oxygen species (ROS) during oxidative stress, were unchanged in leaves but declined in senescing siliques (Fig. 6e).

**Figure 7:**
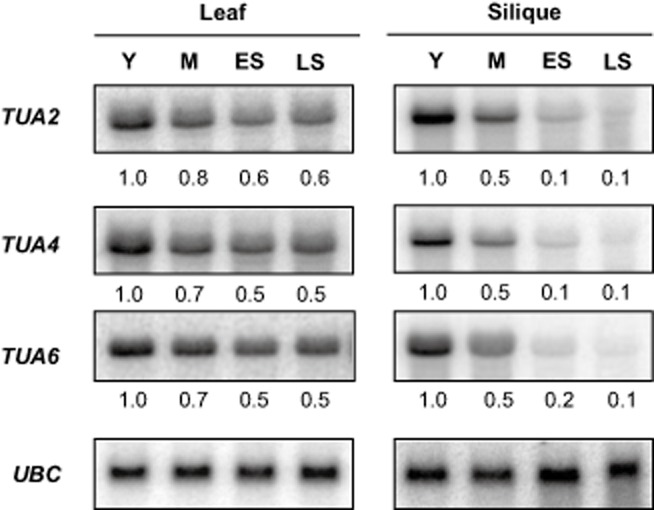
Senescence regulation of sen-sRNA3 targets. Northern blots showing differential expression of sen-sRNA3 targets in senescing leaf and silique. Data is representative of at least two biological replicates. Stages were selected as described in Fig. 1. *UBC* (*At5g25760*) normalized quantification values. One representative *UBC* is shown.

Targets of sen-sRNA3 include three members of the alpha tubulin family, *TUA2*, *TUA4* and *TUA6* (Fig. 4c), which were examined via northern blotting. All three of these targets declined to approximately half of their normal expression level in late senescing leaves (Fig. 7). In siliques, this decline was substantially stronger, with all targets of sen-sRNA3 declining to nearly undetectable levels by late senescence (Fig. 7).

## Discussion

In this study, we report the first sRNA and PARE libraries from senescing leaves and siliques, which enabled large-scale examination of miRNA expression and miRNA target cleavage in these tissues. Seven previously annotated miRNAs were demonstrated to change as senescence progressed, in a tissue-specific manner. Deep sequencing data also enabled identification of a new regulated sRNA locus, the *sen-sRNA* locus, which is most strongly expressed in senescing leaves and siliques (Fig. 4). Examination of the expression levels and decay intermediates of the targets of these known miRNAs and the new sen-sRNAs revealed that they also undergo tissue-specific changes during the course of senescence. Instances of coherent and incoherent regulation were observed between the miRNAs and their targets, a number of which are associated with nutrient limitation.

### Differential expression of miRNAs and their target mRNAs in leaves and siliques

It has been reported that a significant change in the levels of both micro- and macronutrients occurs in senescing leaves (Bleecker & Patterson 1997; Gan & Amasino 1997; Himelblau & Amasino 2001). These changes are thought to be the result of a mobilization of resources away from vegetative tissue and towards developing reproductive tissue and lead to a host of expression changes in both tissues (Leopold 1961; Guiboileau *et al*. 2010). Our analysis of miRNA expression during the course of senescence revealed different, and often completely opposite, expression patterns between leaves and siliques (Figs. 2 & 3). In keeping with the nutrient remobilization hypothesis, five out of the seven miRNAs (miR408, miR399, miR827, miR447 and miR398), which were senescence regulated, have been previously reported to be nutrient responsive (Fujii *et al*. 2005; Abdel-Ghany & Pilon 2008; Dugas & Bartel 2008; Pant *et al*. 2009; Kant *et al*. 2011), indicating that they may play a currently underappreciated role in controlling the flow of nutrients from vegetative to reproductive tissue.

miR399 and miR827 were both strongly induced during senescence in leaves but showed more complex pattern of regulation in siliques, where their expression was initially lower in early senescence (Fig. 2). The correlation between these two miRNAs in both leaves and siliques is consistent with their reported response to phosphate levels (Kant *et al*. 2011). However, their target genes *PHO2* and *NLA*, which are both negative repressors of phosphate transport, responded differently to senescence than they do to phosphate deficiency (Fig. 6b,c; Fujii *et al*. 2005). In leaves, *PHO2* gradually increased during senescence, showing incoherent regulation with miR399 and an opposite expression change than has been reported in non-senescence-induced phosphate deficiency. However, it has been shown that while miR399 and *PHO2* are co-expressed in vascular tissue, miR399 alone is expressed in mesophyll cells, where it may have a currently unidentified target (Aung *et al*. 2006), which could be repressed during senescence. Additionally, *Arabidopsis* expresses target mimics that sequester miR399, which could explain how both miR399 and *PHO2* levels could increase under the same conditions (Franco-Zorrilla *et al*. 2007). The miR827 target *NLA* also increased in senescing leaves, showing incoherent regulation and an opposite pattern than has been reported in phosphate deficient *Arabidopsis*. However, both PARE (data not shown) and northern blot (Fig. 6c) showed an even stronger accumulation of the miR827 directed cleavage product, which is consistent with elevated target and miRNA levels. Given *NLA's* role in regulating phosphate transport in a nitrate-dependent manner, it is likely that miR827 may be functioning to fine tune this relationship during senescence instead of acting as an on/off switch. Additionally, *NLA* has been shown to positively affect anthocyanin production, which is a well-known senescence phenotype (Peng *et al*. 2007). The incoherent regulation of miR399 and miR827's target genes, which does not occur under whole-plant nutrient starvation, implies that nutrient-responsive miRNAs and their target genes may play additional roles in adaptation to shifting nutrient balance during natural senescence.

miR447, which has been shown to be induced under nitrate-deficient conditions (Pant *et al*. 2009), was mildly increased during senescence in leaves (Fig. 2), where it is known that nitrate levels decrease with age (Diaz *et al*. 2008). Its target *2PGK*, however, has been reported to be strongly silique and flower preferential and was undetectable in leaves at any stage. In siliques, miR447 had the lowest level of expression in early senescence where its target *2PGK* showed its highest expression (Fig. 6). The increase of *2PGK* as siliques transitioned from young to early senescence is consistent with earlier findings, which implicate increases of this gene in the silique maturation-induced accumulation of phytic acid, a phosphate storage compound found in seeds, which is inaccessible to human absorption and considered an anti-nutrient (Stevenson-Paulik *et al*. 2005). Knockouts of *2PGK* have been shown to have reduced phytic acid, a desirable agricultural trait, but also have reduced seed germination under stress (Kim & Tai 2011). The natural repression of *2PGK* by miR447, which is shown to be reduced in late senescence, may be more amenable to manipulation in future work.

Of the five nutrient-responsive miRNAs, which changed during the course of aging, only miR398 responded in an opposite manner in senescence compared with its demonstrated response to nutrient deficiency. While sucrose, phosphate and nitrate deficiencies have been reported to repress miR398 (Dugas & Bartel 2008; Pant *et al*. 2009), its levels were actually increased during aging in leaves. Surprisingly *COX5b-1*, a target of miR398, was unchanged during senescence (Fig. 6e) despite its reported responses to sucrose levels (Comelli *et al*. 2009). Other targets of miR398, *CSD1* and *CSD2*, however, were down-regulated during senescence in siliques. This is surprising, given the reduction of miR398 during senescence in siliques (Fig. 2) and the known increase in ROS during senescence (Woo *et al*. 2004) and indicates that the role of miR398 in senescence warrants further investigation. Like miR399 and miR827, this miRNA may also play a different role in senescence than it does in whole-plant nutrient deficiency and oxidative stress.

The majority of senescence-regulated miRNAs have previously been reported to respond to changes in nutrient levels. However, members of the miR156/157 family and miR396a-3p have not been shown to change significantly under nutrient starvation. miR396a itself has been reported to target the *GROWTH REGULATING TRANSCRIPTION FACTOR* (*GRF*) family (Jones-Rhoades & Bartel 2004), but increased very slightly in senescing leaves and siliques. Interestingly, the miR396a precursor was recently reported to produce an abundant miR star, miR396a-5p (Jeong *et al*. 2013), which was induced during senescence in leaves and repressed in siliques (Fig. 3b). This miRNA is currently without any validated targets, making it difficult to speculate on its potential role in senescence. All members of the miR156/157 family are predicted to target members of the *SPL* transcription factor family, which control development. Despite overlapping predicted targets, it has recently been reported that miR156 and miR157 family members do mediate differential cleavage of specific SPL transcripts (Meng *et al*. 2012). The senescence repression of miR157 in leaves without any change in miR156 therefore may be a fine-tuning mechanism to control expression of specific SPLs as senescence progresses in leaves. In siliques, both miRNAs were strongly induced in late senescence, which could lead to repression of multiple SPL family members in this tissue.

miR408 targets genes involved in many different biological processes and its senescence regulation could potentially have a variety of effects. Although *UCC2* and *PLANTACYANIN* are both validated targets of miR408, they showed incoherent regulation by increasing as senescence progressed (Fig. 6), and are involved in processes, which have no known involvement in the senescence process (Dong *et al*. 2005). In contrast, two other targets, *CUPREDOXIN* and *LAC3*, both showed the expected reduction, which correlated with an increase of miR408 during senescence (Fig. 6). *CUPREDOXIN* has been proposed to be involved in xylan biosynthesis (Mutwil *et al*. 2009) and its down-regulation by miR408 could therefore affect cell wall integrity during senescence. Similarly *LAC3*, which was also reduced during senescence, is thought to act on the cell wall. Transgenic knockdowns of *LAC3* have been reported to have altered cell wall integrity and more readily detachable xylem fibre cell walls in poplar (Ranocha *et al*. 1999). Senescence is known to bring about a host of cell wall changes (Bleecker & Patterson 1997) and the regulation of *CUPREDOXIN* and *LAC3* by miR408 imply that this miRNA may be playing an important role in the process.

### *Sen-sRNAs* are induced by senescence and target the alpha tubulin gene family

The discovery of the senescence-inducible *sen-sRNA* locus through deep sequencing of sRNAs in senescing tissues highlights the importance of continued investigation of the sRNA transcriptome under diverse sets of conditions. It is interesting that the *sen-sRNA* precursor can form a stem-loop structure, passes strand bias criteria for miRNAs and sRNAs from it guide cleavage of target mRNAs similar to miRNAs. Thus, although sen-sRNAs have miRNA-like function, the *sen-sRNA* precursor fails the abundance cut-offs for precise excision required for formal annotation as a miRNA locus (Meyers *et al*. 2008).

The expression of nearly all members of the alpha tubulin gene family has been reported to decline during senescence (Wagstaff *et al*. 2009; Keech *et al*. 2010; Breeze *et al*. 2011). Intriguingly, we show that all alpha tubulin mRNAs that are sen-sRNA3 targets decline slightly in leaves and drastically in siliques (Fig. 7), which has the potential to lead to massive changes in cellular integrity. sen-sRNA3 may therefore be working in concert with miR408 to alter cellular structure by targeting alpha tubulins and represents an interesting candidate for future studies investigating senescence-associated changes in cell structure. Both miR408 and sen-sRNA3 are the first indications that sRNAs, which have demonstrated roles in many developmental processes (Gandikota *et al*. 2007), are also involved in what is often termed the final developmental stage, senescence.

Taken together, these findings highlight the integral, and currently underappreciated, role that miRNAs play during senescence. The high-percentage senescence-regulated miRNAs, which have previously been noted as nutrient responsive, and their predominantly antagonistic regulation between leaves and siliques, implies they may function in the regulatory pathways, which govern mobilization of nutrients away from vegetative tissue and towards reproductive tissue during the course of senescence. Beyond the mobilization of nutrients, several miRNAs have target genes that could be of strong interest agriculturally, such as miR447, which controls phytic acid accumulation, or miR408 and sen-sRNA3, which have the potential to alter cell structure and integrity. This study greatly expands upon the existing knowledge of the roles of miRNAs in senescence and provides a number of new avenues to explore.

## Supporting Information

**Figure S1.** Small RNA size distribution plots. Genome-matched small RNA data were normalized to transcripts per two million (TP2M) and the total abundance of each size class of small RNA was plotted. (A) Size distributions in young, mature, early senescing and late senescing leaf. (B) Size distributions in young, mature, early senescing and late senescing silique. **Figure S2.** Comparison of miRNA abundance between mature and other stages. miRNA abundance comparison between mature and other stages. Correlation coefficients for each comparison are also shown. (A) Mature leaves compared with young leaves, early senescing leaves and late senescing leaves. (B) Mature siliques compared with young siliques, early senescing siliques and late senescing siliques. **Table S1.** Oligomers used in this study. **Table S2.** miRNA quantification from leaf and silique small RNA libraries. Values represent the abundance of the miRNA sequence in the indicated library (abbreviations as in Table 1) normalized to TP2M (transcripts per two million) trimmed genome-matched reads.
