# Supplementary material for: Differential expression of miRNAs and their target genes in senescing leaves and siliques: insights from deep sequencing of small RNAs and cleaved target RNAs
Source: Plant Cell Environ. 2014 Aug 13;38(1):188–200. doi: 10.1111/pce.12393 (PMC4304344; doi:10.1111/pce.12393)
Supplement: Supplementary file 1 — Figure S1. Small RNA size distribution plots. Genome-matched small RNA data were normalized to transcripts per two million (TP2M) and the total abundance of each size class of small RNA was plotted. (A) Size distributions in young, mature, early senescing and late senescing leaf. (B) Size distributions in young, mature, early senescing and late senescing silique. Figure S2. Comparison of miRNA abundance between mature and other stages. miRNA abundance comparison between mature and other stages. Correlation coefficients for each comparison are also shown. (A) Mature leaves compared with young leaves, early senescing leaves and late senescing leaves. (B) Mature siliques compared with young siliques, early senescing siliques and late senescing siliques. Table S1. Oligomers used in this study. Table S2. miRNA quantification from leaf and silique small RNA libraries. Values represent the abundance of the miRNA sequence in the indicated library (abbreviations as in Table 1) normalized to TP2M (transcripts per two million) trimmed genome-matched reads. [file pce0038-0188-sd1.pdf]

SUPPORTING INFORMATION

Supplemental Table S1. Oligomers used in this study

Primers used to amplify gene-specific probe

**Name**	**Sequence**
2PGK-Forward	AGCTCGCCTAGGATGTCTGA
2PGK-Reverse	CATGGGGCACCCAAGAGAAT
AT1G72230-Forward	GAATGGTCGCTCGGGAAAGA
AT1G72230-Reverse	CCAAAGCATCCAACGGCTTC
COX5B-1-Forward	ACATTTGGGGAGTTTCTTCGC
COX5B-1-Reverse	GGGATAACGAAAGAAGATGCGG
CSD1-Forward	AGGCCAAGTAACAATGGCGA
CSD1-Reverse	CAGCTTTAGCCCTGGAGACC
CSD2-Forward	CCAAACGTCAAACATAGCAGCA
CSD2-Reverse	GGGTTGAAATGTGGTCCTGTTG
LAC3-Forward	CCGTTCGACAACACAACCAC
LAC3-Reverse	TTCTCTGGCGTGACAATGCT
NLA-Forward	GCCGTCGCCATTCGAAAAAT
NLA-Reverse	TGCTTCTGCGGTTTTCAAGC
PHO2-Forward	ACCGTTTCTCATCAAGGCGT
PHO2-Reverse	GGTGACAGAGACACGCTCAA
PLANTACYANIN-Forward	GTACACGGTCGGTGACTCTG
PLANTACYANIN-Reverse	GAGACCGCACCGGATACTTT
TUA2-Forward	CTGAGGTTCGATGGTGCCTT
TUA2-Reverse	CTACCGCCACAAGCAAGAGA
TUA4-Forward	GCGTCTTCATAAACGCCCTTC
TUA4-Reverse	ACAGCGTTGAAGACGAGGAA
TUA6-Forward	ACAGCATCAAACACCTTCCTCA
TUA6-Reverse	TGTAATGACCACGGGCGAAA

Oligonucleotides used to detect sRNAs

**Name**	**Sequence**
miR156a-g	GTGCTCACTCTCTTCTGTCA
miR157	GTGCTCTCTATCTTCTGTCAA
miR164	TGCACGTGCCCTGCTTCTCCA
miR398	AAGGGGTGACCTGAGAACACA
miR399	CAGGGCAAATCTCCTTTGGCA
miR408	GCCAGGGAAGAGGCAGTGCAT
miR447	CAACAAAACATCTCGTCCCCAA
miR827	AGTTTGTTGATGGTCATCTAA
sen-sRNA 1-2	TTTTCTTCCAAGGTTCAAGAA
sen-sRNA 3-4	GATCCGTTCTTGAACCTTGCA
sen-sRNA5	AAACCCTTCACTTTGTCGAAT

Bridge oligonucleotides to detect miRNAs

**Name**	**Bridge oligo sequence**
miR396a	GAATGTCATAAGCGCAGTTCAAGAAAGCTGTGGAA
miR396a*	GAATGTCATAAGCGCTTCCCACAGCTTTATTGAAC

Oligonucleotides used to make small RNA libraries

**Name**	**Bridge oligo sequence**
5’ RNA adapter	GUUCAGAGUUCUACAGUCCGACGAUC
3’ RNA adapter *	UCGUAUGCCGUCUUCUGCUUG
RT primer	CAAGCAGAAGACGGCATACGA
Forward PCR primer	AATGATACGGCGACCACCGACAGGTTCAGAGTTCTACAGTCCGA
Reverse PCR primer	CAAGCAGAAGACGGCATACGA

Supplemental Table S2. miRNA quantification from leaf and silique small RNA libraries.

Values represent the abundance of the miRNA sequence in the indicated library (abbreviations as in Table 1) normalized to TP2M (transcripts per two million) trimmed genome-matched reads.

miRNA	miRNA sequence	C0LY	C0LM	C0LSeE	C0LSeL	C0SilY	C0SilM	C0SilES	C0SilLS
ath-miR156a-f	UGACAGAAGAGAGUGAGCAC	247,907	307,892	117,878	362,175	2,704	10,728	4,000	4,865
ath-miR156g	CGACAGAAGAGAGUGAGCAC	143	301	96	298	1	5	1	1
ath-miR156h	UGACAGAAGAAAGAGAGCAC	76	170	54	121	107	81	9	2
ath-miR156i	UGACAGAAGAGAGAGAGCAG	0	0	0	0	0	0	0	0
ath-miR156j	UGACAGAAGAGAGAGAGCAC	264	222	219	340	2	7	2	4
ath-miR157a-c	UUGACAGAAGAUAGAGAGCAC	582,372	770,809	398,060	187,224	154	76	18	12
ath-miR157d	UGACAGAAGAUAGAGAGCAC	47,761	68,392	25,909	24,151	9	6	1	1
ath-miR158a	UCCCAAAUGUAGACAAAGCA	9,240	9,345	3,420	5,016	23,727	21,819	10,342	9,868
ath-miR158b	CCCCAAAUGUAGACAAAGCA	4	7	1	3	44	22	5	4
ath-miR159a	UUUGGAUUGAAGGGAGCUCUA	370	267	123	458	8,856	8,803	4,064	12,020
ath-miR159b	UUUGGAUUGAAGGGAGCUCUU	69	41	22	64	9,345	11,766	1,547	1,678
ath-miR159c	UUUGGAUUGAAGGGAGCUCCU	0	0	0	0	10	6	2	2
ath-miR160a-c	UGCCUGGCUCCCUGUAUGCCA	86	81	215	70	4	3	2	3
ath-miR161.1	UGAAAGUGACUACAUCGGGGU	301	377	335	228	1,021	884	282	302
ath-miR161.2	UCAAUGCAUUGAAAGUGACUA	970	497	1,248	988	155	219	89	113
ath-miR162a-d	UCGAUAAACCUCUGCAUCCAG	643	402	111	178	960	743	560	891
ath-miR163	UUGAAGAGGACUUGGAACUUCGAU	123	24	126	475	587	519	233	373
ath-miR164a-b	UGGAGAAGCAGGGCACGUGCA	5,680	5,485	2,149	1,049	2	1	0	0
ath-miR164c	UGGAGAAGCAGGGCACGUGCG	631	1,009	1,123	1,047	0	0	0	0
ath-miR165a-g	UCGGACCAGGCUUCAUCCCCC	1,019	315	238	530	48,667	30,592	23,711	11,641
ath-miR167a-b	UGAAGCUGCCAGCAUGAUCUA	102,116	124,412	226,822	151,561	560	394	91	135
ath-miR167c	UAAGCUGCCAGCAUGAUCUUG	1	2	0	0	0	1	0	0
ath-miR167d	UGAAGCUGCCAGCAUGAUCUGG	3,912	2,604	2,917	3,815	315	343	125	490
ath-miR168a-b	UCGCUUGGUGCAGGUCGGGAA	32,540	65,832	21,598	20,144	2,448	805	420	292
ath-miR169a	CAGCCAAGGAUGACUUGCCGA	43	40	38	6	3	2	0	0
ath-miR169b-f	CAGCCAAGGAUGACUUGCCGG	1	2	6	7	0	0	0	0
ath-miR169g-3p	UCCGGCAAGUUGACCUUGGCU	1	0	0	0	0	0	0	0
ath-miR169g-5p	UGAGCCAAGGAUGACUUGCCG	41	46	66	116	3	2	0	0
ath-miR169h-n	UAGCCAAGGAUGACUUGCCUG	26	6	7	3	22	18	2	2
ath-miR170	UGAUUGAGCCGUGUCAAUAUC	3	0	0	5	36	27	9	16
ath-miR171a	UGAUUGAGCCGCGCCAAUAUC	31	20	18	17	126	59	27	60
ath-miR171b-c	UUGAGCCGUGCCAAUAUCACG	7	7	2	3	30	20	4	4
ath-miR172a-b	AGAAUCUUGAUGAUGCUGCAU	22,101	19,377	38,208	37,530	24	8	2	2
ath-miR172b-5p	GCAGCACCAUUAAGAUUCAC	7	7	15	9	0	0	0	0
ath-miR172c-d	AGAAUCUUGAUGAUGCUGCAG	3	4	8	43	162	58	8	5
ath-miR172e	GGAAUCUUGAUGAUGCUGCAU	43	19	49	71	3	1	0	0
ath-miR173-3p	UGAUUCUCUGUGUAAGCGAAA	18	14	48	38	2	1	1	1

ath-miR173-5p	UUCGCUUGCAGAGAGAAAUCAC	1,836	1,706	5,695	2,520	110	68	5	12
ath-miR1886.1	UGAGAGAAGUGAGAUGAAAUC	2	1	3	0	3	2	1	0
ath-miR1886.2	UGAGAUGAAAUCUUUGAUUGG	52	48	77	39	74	77	24	19
ath-miR1886.3	AAUUAAAGAUUUCAUCUUACU	2	2	3	23	2	2	1	1
ath-miR1887	UACUAAGUAGAGUCUAAGAGA	0	0	0	0	0	0	0	0
ath-miR1888a	UAAGUUAAGAUUUGUGAAGAA	10	9	16	8	1	1	0	0
ath-miR1888b	UUAGGCUAAGAUUUGUGAAGA	1	0	0	0	0	0	0	0
ath-miR2111a-3p	GUCCUCGGGAUGCGGAUUACC	0	0	0	0	2	0	0	0
ath-miR2111a-5p	UAAUCUGCAUCCUGAGGUUUA	0	0	0	0	0	0	0	0
ath-miR2111b-3p	AUCCUCGGGAUACAGUUUACC	2	1	8	6	2	1	1	4
ath-miR2112-3p	CUUUAUAUCCGCAUUUGCGCA	2	3	0	0	1	0	0	0
ath-miR2112-5p	CGCAAAUGCGGAUAUCAAUGU	0	1	2	11	7	4	1	2
ath-miR2933a-b	GAAAUCGGAGAGGAAAUUCGCC	0	0	0	0	0	0	0	0
ath-miR2934-3p	CAUCCAAGGUGUUUGUAGAAA	0	0	0	0	0	0	0	0
ath-miR2934-5p	UCUUUCUGCAAACGCCUUGGA	0	0	0	0	0	0	0	0
ath-miR2936	CUUGAGAGAGAGAACACAGACG	0	1	3	0	0	0	0	0
ath-miR2937	AUAAGAGCUGUUGAAGGAGUC	0	0	0	0	0	0	0	0
ath-miR2938	GAUCUUUUGAGAGGGUUCCAG	0	0	0	0	0	0	0	0
ath-miR2939	UAACGCACAACACUAAGCCAU	0	0	0	0	0	0	0	0
ath-miR319a-b	UUGGACUGAAGGGAGCUCCCU	1	0	0	0	1,464	1,839	1,038	1,323
ath-miR319c	UUGGACUGAAGGGAGCUCCUU	0	0	1	0	192	213	95	146
ath-miR3434-3p	UCAGAGUAUCAGCCAUGUGA	0	0	0	0	0	0	0	0
ath-miR3434-5p	ACUUGGCUGAUUCUAUUAUU	0	0	0	0	2	1	0	1
ath-miR3440b-3p	UGGAUUGGUCAAGGGAAGCGU	2	2	0	0	0	0	0	0
ath-miR3440b-5p	UUUUCUUGGCCCAUCCACUUC	0	0	0	0	0	0	0	0
ath-miR390a-b	AAGCUCAGGAGGGAUAGCGCC	151	157	237	79	569	326	578	651
ath-miR391	UUCGCAGGAGAGAUAGCGCCA	6,241	9,629	9,806	9,286	7	3	1	0
ath-miR3932a-b	AACUUUGUGAUGACAACGAAG	2	5	0	8	0	0	0	0
ath-miR3933	AGAAGCAAAAUGACGACUCGG	2	3	9	0	0	0	0	0
ath-miR393a-b	UCCAAAGGGAUCGCAUUGAUCC	31	24	13	40	206	80	34	47
ath-miR394a-b	UUGGCAUUCUGUCCACCUCC	6	2	0	5	274	226	95	127
ath-miR395a-f	CUGAAGUGUUUGGGGGAACUC	4	6	0	13	1	2	0	1
ath-miR396a	UUCCACAGCUUUCUUGAACUG	855	582	424	507	319	127	267	666
ath-miR396b	UUCCACAGCUUUCUUGAACUU	38	26	36	31	5,475	2,770	548	345
ath-miR397a	UCAUUGAGUGCAGCGUUGAUG	2	3	4	0	4	1	0	0
ath-miR397b	UCAUUGAGUGCAUCGUUGAUG	61	47	27	13	58	28	8	15
ath-miR398a	UGUGUUCUCAGGUCACCCCUU	0	0	0	0	1	0	0	0
ath-miR398b-c	UGUGUUCUCAGGUCACCCCUG	4	10	3	21	12,281	11,472	1,005	300
ath-miR399a	UGCCAAAGGAGAUUUGCCCUG	0	0	1	0	30	15	1	1
ath-miR399b-c	UGCCAAAGGAGAGUUGCCCUG	8	5	14	23	127	82	12	7
ath-miR399d	UGCCAAAGGAGAUUUGCCCCG	0	0	0	0	0	0	0	0

ath-miR399e	UGCCAAAGGAGAUUUGCCUCG	0	0	0	0	0	0	0	0
ath-miR399f	UGCCAAAGGAGAUUUGCCCGG	1	2	8	7	1	0	0	0
ath-miR400	UAUGAGAGUAUUAUAAGUCAC	21	19	75	100	41	45	18	21
ath-miR401	CGAAACUGGUGUCGACCGACA	0	0	0	0	0	0	0	0
ath-miR402	UUCGAGGCCUAUUAAACCUCUG	3	2	0	10	0	1	0	0
ath-miR403	UUAGAUUCACGCACAAACUCG	192	115	107	105	475	493	443	814
ath-miR404	AUUAACGCUGGCGGUUGCGGCAGC	0	0	0	0	0	0	0	0
ath-miR405a,b,d	AUGAGUUGGGUCUAACCCAUAACU	0	0	0	0	0	0	0	0
ath-miR406	UAGAAUGCUAUUGUAAUCCAG	0	0	0	0	0	0	0	0
ath-miR407	UUUAAAUCAUAUACUUUUGGU	0	0	0	0	0	0	0	0
ath-miR408	AUGCACUGCCUCUUCCCUGGC	190	318	607	454	235	138	95	239
ath-miR413	AUAGUUUCUCUUGUUCUGCAC	0	0	0	0	0	0	0	0
ath-miR414	UCAUCUUCAUCAUCAUCGUCA	0	0	0	0	0	0	0	0
ath-miR415	AACAGAGCAGAAACAGAACAU	0	0	0	0	0	0	0	0
ath-miR416	GGUUCGUACGUACACUGUUCA	0	0	0	0	0	0	0	0
ath-miR417	GAAGGUAGUGAAUUUGUUCGA	0	0	0	0	0	0	0	0
ath-miR418	UAAUGUGAUGAUGAACUGACC	0	0	0	0	0	0	0	0
ath-miR419	UUAUGAAUGCUGAGGAUGUUG	0	0	0	0	0	0	0	0
ath-miR420	UAAACUAAUCACGGAAAUGCA	0	0	0	0	0	0	0	0
ath-miR4221	UUUUCCUCUGUUGAAUUCUUGC	0	0	0	0	0	0	0	0
ath-miR4227	UCACUGGUACCAAUCAUUCCA	0	0	0	0	0	0	0	0
ath-miR4228	AUAGCCUUGAACGCCGUCGUU	0	0	0	0	0	0	0	0
ath-miR4239	UUUGUUAUUUUCGCAUGCUCC	0	0	0	0	0	0	0	0
ath-miR4240	UGACUAGACCCGUAACAUUAC	0	0	0	0	0	0	0	0
ath-miR4243	UUGAAAUUGUAGAUUUCGUAC	0	0	0	0	0	0	0	0
ath-miR4245	ACAAAGUUUUAUACUGACAAU	5	3	10	0	1	1	0	0
ath-miR426	UUUUGGAAAUUUGUCCUUACG	0	0	0	0	0	0	0	0
ath-miR447a-3p	UUGGGGACGAGAUGUUUUGUUG	15	8	6	0	2	2	0	0
ath-miR447a.2-3p	UAUGGAAGAAAUUGUAGUAUU	5	2	4	0	265	577	47	26
ath-miR447c-3p	UUGGGGACGACAUCUUUUGUUG	0	0	0	0	0	0	0	0
ath-miR447c-5p	CCCCUUACAAUGUCGAGUAAA	0	0	0	0	0	1	0	0
ath-miR472	UUUUUCCUACUCCGCCCAUACC	1	0	0	0	15	10	2	7
ath-miR5012	UUUUACUGCUACUUGUGUUCC	0	0	0	0	3	2	1	1
ath-miR5013	UUUGUGACAUCUAGGUGCUUU	0	0	0	0	0	0	0	0
ath-miR5014a-3p	UUGUACAAAUUUAAGUGUACG	3	4	4	0	0	0	0	0
ath-miR5014a-5p	ACACUUAGUUUUGUACAACAU	1	1	0	9	0	0	0	0
ath-miR5014b	AUUUGUACACCUAGAUCUGUA	4	3	7	10	0	0	0	0
ath-miR5015	UUGGUGUUAUGUGUAGUCUUC	0	0	0	0	0	0	0	0
ath-miR5016	UUCUUGUGGAUUCCUUGGAAA	0	0	0	0	0	0	0	0
ath-miR5017	UUAUACCAAAUUAAUAGCAAA	0	0	0	0	0	0	0	0
ath-miR5018	UUAAAGCUCCACCAUGAGUCCAAU	0	0	0	0	0	0	0	0

ath-miR5019	UGUUGGGAAAGAAAAACUCUU	0	0	0	0	0	0	0	0
ath-miR5020a	UGGAAGAAGGUGAGACUUGCA	3	1	0	0	0	0	0	0
ath-miR5020b	AUGGCAUGAAAGAAGGUGAGA	152	393	218	75	1	1	0	0
ath-miR5020c	UGGCAUGGAAGAAGGUGAGAC	1	2	2	0	0	0	0	0
ath-miR5021	UGAGAAGAAGAAGAAGAAAA	0	0	0	0	0	0	0	0
ath-miR5022	GUCAUGGGGUAUGAUCGAAUG	0	0	0	0	0	0	0	0
ath-miR5023	AUUGGUAGUGGAUAAGGGGGC	0	1	0	0	0	0	0	0
ath-miR5024-3p	CCGUAUCUUGGCCUUGUCAUU	0	0	0	0	2	1	1	1
ath-miR5024-5p	AUGACAAGGCCAAGAUAUAACA	0	0	0	1	0	0	0	0
ath-miR5025	ACUGUAUAUAUGUAAGUGACA	0	0	0	0	0	0	0	0
ath-miR5026	ACUCAUAAGAUCGUGACACGU	5,934	7,542	2,899	3,863	4	5	2	4
ath-miR5027	ACCGGUUGGAACUUGCCUUAA	0	0	0	0	0	0	0	0
ath-miR5028	AAUUGGGUUUAUGCUAGAGUU	0	0	0	0	1	1	0	0
ath-miR5029	AAUGAGAGAGAACACUGCAAA	0	0	0	0	1	2	0	0
ath-miR5595a-b	ACAUAUGAUCUGCAUCUUUGC	0	1	0	0	19	12	0	0
ath-miR5628	GAAAUAGCGAAGAUAUGAUUA	0	0	0	0	0	0	0	0
ath-miR5629	UUAGGGUAGUUAACGGAAGUUA	2	1	0	0	0	0	0	0
ath-miR5630a-b	GCUAAGAGCGGUUCUGAUGGA	0	0	0	0	0	0	0	0
ath-miR5631	UGGCAGGAAAGACAUAAUUUU	0	0	0	0	0	0	0	0
ath-miR5632	UUGGAUUUAUAGUUGGAUAAG	0	0	0	0	0	0	0	0
ath-miR5633	UAUGAUCAUCAGAAAACAGUG	1	1	0	0	0	0	0	0
ath-miR5634	AGGGACUUUGUGAAUUUAGGG	1	0	0	6	0	0	0	0
ath-miR5635a-d	UGUUAAGGAGUGUUAACGGUG	2	2	0	0	0	0	0	0
ath-miR5636	CGUAGUUGCAGAGCUUGACGG	2	1	0	0	0	0	0	0
ath-miR5637	AAUGCGCAACUCUAUAUUUCC	0	0	0	0	1	1	0	0
ath-miR5638a	AUACCAAAACUCUCUCACUUU	0	0	0	0	0	0	0	0
ath-miR5638b	ACAGUGGUCAUCUGGUGGGCU	0	0	0	0	3	5	1	1
ath-miR5639	UAGUCCACUGUGGUCUAAGGC	2	1	2	3	2	1	0	1
ath-miR5640	UGAGAGAAGGAAUUAGAUUCA	1	1	2	0	2	2	0	0
ath-miR5641	UGGAAGAAGAUGAUAGAAUUA	0	0	0	7	0	0	0	0
ath-miR5642a-b	UCUCGCGCUUGUACGGCUUU	16	19	3	1	1	1	0	0
ath-miR5643a-b	AGGCUUUUAAGAUCUGGUUGC	4	4	9	0	1	0	0	0
ath-miR5644	GUGGGUUGCGGAUAACGGUA	1	2	1	0	0	0	0	0
ath-miR5645a-b	AUUUGAGUCAUGUCGUUAAG	0	0	0	0	0	0	0	0
ath-miR5645c	AACCUAUUUAACGACAUGACU	0	0	0	0	0	0	0	0
ath-miR5646	GUUCGAGGCACGUUGGGAGG	0	0	0	0	0	0	0	0
ath-miR5647	UCAAGUUUGAUGACGAUUCCA	1	1	0	0	0	0	0	0
ath-miR5648-3p	AUCUGAAGAAAAUAGCGGCAU	22	30	52	0	0	0	0	0
ath-miR5648-5p	UUUGGAAAUAUUUGGCUUGACU	9	3	9	8	1	0	0	0
ath-miR5649a-b	AUUGAAUAUGUUGGUUACUAU	0	0	0	0	0	0	0	0
ath-miR5650	UUGUUUUGGAUCUUAGAUACA	0	0	0	0	1	7	4	1

ath-miR5651	UUGUGCGGUUCAAAUAGUAAC	3	6	4	6	12	18	7	5
ath-miR5652	UUGAAUGUGAAUGAAUCGGGC	7	6	6	0	0	0	0	1
ath-miR5653	UGGGUUGAGUUGAGUUGAGUUGGC	4	1	5	1	0	1	2	1
ath-miR5654-3p	UGGAAGAUGCUUUGGGAUUUAUU	11	5	25	15	0	0	0	0
ath-miR5654-5p	AUAAAUCCCAACAUCUUCCA	0	1	1	0	0	0	0	0
ath-miR5655	AAGUAGACACAUAAGAAGGAG	1	2	5	8	0	0	0	0
ath-miR5656	ACUGAAGUAGAGAUUGGGUUU	4	4	15	6	0	0	0	0
ath-miR5657	UGGACAAGGUUAGAUUUGGUG	0	1	0	0	0	0	0	0
ath-miR5658	AUGAUGAUGAUGAUGAUGAAA	0	0	0	0	0	0	0	0
ath-miR5659	CGAUGAAGGUCUUUGGAACGGUA	22	7	16	37	0	0	1	0
ath-miR5660	CAGGUGGUUAGUGCAAUGGAA	0	0	0	0	0	0	0	0
ath-miR5661	AGAGGUACAUCAUGUAGUCUG	5	5	0	7	0	0	0	0
ath-miR5662	AGAGGUGACCAUUGGAGAUG	0	0	0	0	0	0	0	0
ath-miR5663	AGCUAAGGAUUUGCAUUCUCA	13	11	17	7	1	1	0	0
ath-miR5664	AUAGUCAAUUUUAUCGGUCUG	1	1	3	0	0	0	0	0
ath-miR5665	UUGGUGGACAAGAUCUGGGAU	0	0	0	0	0	0	0	0
ath-miR5666	AUGGGACAUCGAGCAUUUAAU	0	0	0	0	0	1	0	0
ath-miR5996	UGACAUCCAGAUAGAAGCUUUG	112	81	71	71	0	0	0	0
ath-miR5997	UGAAACCAAGUAGCUAAAUAG	2	1	11	7	0	0	0	0
ath-miR5998a-b	ACAGUUUGUGUUUUGUUUUGU	0	0	4	0	0	0	0	0
ath-miR5999	UCUUCACUAUUAGACGGACAA	5	4	0	10	0	0	0	0
ath-miR771	UGAGCCUCUGUGGUAGCCCUCA	0	0	0	0	11	2	0	0
ath-miR773a	UUUGCUUCCAGCUUUUGUCUC	0	0	0	0	0	0	0	0
ath-miR773b-3p	UUUGAUUCCAGCUUUUGUCUC	0	0	0	0	0	0	0	0
ath-miR773b-5p	GGCAAUAACUUGAGCAAACA	0	0	0	0	0	0	0	0
ath-miR774a	UUGGUUACCCAUAUGGCCAUC	0	0	0	0	5	1	0	0
ath-miR774b-3p	CAUCCAUAUUUUCAUCUCGAA	0	0	0	0	0	0	0	0
ath-miR774b-5p	UGAGAUGAAGAUAUGGGUGAU	0	0	0	0	0	0	0	0
ath-miR775	UUCGAUGUCUAGCAGUGCCA	8	16	10	7	34	25	4	3
ath-miR776	UCUAAGUCUUCUAUUGAUGUU	0	0	0	0	0	0	0	0
ath-miR777	UACGCAUUGAGUUUCGUUGCUU	3	1	5	14	60	26	3	3
ath-miR778	UGGCUUGGUUUAUGUACACCG	0	0	0	0	0	0	0	0
ath-miR779.1	UUCUGCUAUGUUGCUGCUCAU	0	0	0	0	0	0	0	0
ath-miR779.2	UGAUUGGAAAUUUCGUUGACU	0	1	0	6	31	40	19	17
ath-miR780.1	UCUAGCAGCUGUUGAGCAGGU	0	0	0	0	2	1	0	0
ath-miR780.2	UUCUUCGUGAAUAUCUGGCAU	0	0	0	0	17	31	1	2
ath-miR781a-b	UUAGAGUUUUCUGGAUACUUA	2	1	0	0	46	56	12	14
ath-miR782	ACAAACACCUUGGAUGUUCUU	0	0	0	0	0	0	0	0
ath-miR822	UGCGGGAAGCAUUUGCACAUG	492	396	456	189	12	8	1	0
ath-miR823	UGGGUGGUGAUCAUAUAAGAU	11	16	32	30	4	4	2	2
ath-miR824	UAGACCAUUUGUGAGAAGGGA	342	484	926	483	33	52	30	99

ath-miR825	UUCUCAAGAAGGUGCAUGAAC	203	276	193	97	1	2	1	1
ath-miR826	UAGUCCGGUUUUGGAUACGUG	0	2	1	16	2	2	5	11
ath-miR827	UUAGAUGACCAUCAACAAACU	165	187	520	470	197	334	62	15
ath-miR828	UCUUGCUUAAAUGAGUAUUCCA	0	0	0	0	1	1	0	0
ath-miR829.1	AGCUCUGAUACCAAAUGAUGGAAU	1	0	1	9	6	2	4	6
ath-miR829.2	CAAAUUAAAGCUUCAAGGUAG	0	0	0	0	0	0	0	0
ath-miR830-3p	UAACUAUUUUGAGAAGAAGUG	0	2	17	0	1	0	0	1
ath-miR830-5p	UCUUCUCCAAAUAGUUUAGGUU	0	0	1	0	2	1	0	1
ath-miR831	UGAUCUCUUCGUACUCUUCUUG	0	0	0	0	1	1	0	0
ath-miR832-3p	UUGAUUCCCAAUCCAAGCAAG	0	0	0	0	3	1	0	0
ath-miR832-5p	UGCUGGGAUCGGGAAUCGAAA	0	0	0	0	4	1	1	0
ath-miR833a-3p	UAGACCGAUGUCAACAAACAAG	4	1	3	0	1	0	0	0
ath-miR833a-5p	UGUUUGUUGUACUCGGUCUAGU	0	0	0	5	2	1	1	1
ath-miR833b	UGUUUGUUGACAUCGGUCUAG	6	4	1	0	0	0	0	0
ath-miR834	UGGUAGCAGUAGCGGUGGUAA	1	2	2	0	0	0	0	0
ath-miR835-3p	UGGAGAAGAUACGCAAGAAAG	1	1	0	0	0	0	0	0
ath-miR835-5p	UUCUUGCAUAUGUUCUUUAUC	0	0	0	0	0	0	0	0
ath-miR836	UCCUGUGUUUCCUUUGAUGCGUGG	0	0	0	0	0	0	0	0
ath-miR837-3p	AAACGAACAAAAAACUGAUGG	2	3	3	0	0	0	0	0
ath-miR837-5p	AUCAGUUUCUUGUUCGUUUCA	0	0	0	0	0	0	0	0
ath-miR838	UUUUCUUCUACUUCUUGCACA	0	0	0	0	0	0	0	0
ath-miR839	UACCAACCUUUCAUCGUUCCC	0	0	0	0	2	1	1	0
ath-miR840	ACACUGAAGGACCUAAACUAAC	2	2	3	28	20	13	2	1
ath-miR841a	UACGAGCCACUUGAAACUGAA	102	62	21	28	6	24	11	10
ath-miR841b-3p	CAAUUUCUAGUGGGUCGUAUU	4	2	3	0	0	0	0	0
ath-miR841b-5p	UACGAGCCACUGGAAACUGAA	15	14	5	6	7	13	4	2
ath-miR842	UCAUGGUCAGAUCCGUCAUCC	7	7	0	13	56	39	3	3
ath-miR843	UUUAGGUCGAGCUUCAUUGGA	124	177	215	177	10	17	3	7
ath-miR844-3p	UUAUAAGCCAUCUUACUAGUU	1	1	0	0	3	2	1	1
ath-miR844-5p	UGGUAAGAUUGCUUAUAAGCU	0	1	3	0	0	0	0	0
ath-miR845a	CGGCUCUGAUACCAAUUGAUG	0	1	1	0	174	117	39	71
ath-miR845b	UCGCUCUGAUACCAAAUUGAUG	0	0	0	0	64	34	8	12
ath-miR846	UUGAAUUGAAGUGCUUGAAUU	5	3	5	6	136	496	170	217
ath-miR847	UCACUCCUCUUCUUCUUGAUG	0	0	0	0	1	1	0	0
ath-miR848	UGACAUGGGACUGCCUAAGCUA	24	18	21	14	9	8	2	1
ath-miR849	UAACUAAACAUUGGUGUAGUA	3	1	3	3	0	0	0	0
ath-miR850	UAAGAUCCGGACUACAACAAAG	6	7	5	8	4	6	3	1
ath-miR851-3p	UGGGUGGCAAACAAAGACGAC	2	1	5	0	0	0	0	0
ath-miR851-5p	UCUCGGUUCGCGAUCCACAAG	0	0	0	0	4	1	0	0
ath-miR852	AAGAUAAGCGCCUUAGUUCUG	1	0	0	0	0	0	0	1
ath-miR853	UCCCCUCUUUAGCUUGGAGAAG	0	0	0	3	11	5	1	0

ath-miR854a-e	GAUGAGGAUAGGGAGGAGGAG	0	0	0	0	0	0	0	0
ath-miR855	AGCAAAAGCUAAGGAAAAGGAA	0	0	0	0	0	0	0	0
ath-miR856	UAAUCCUACCAAUAACUUCAGC	0	0	0	0	0	0	0	0
ath-miR857	UUUUGUAUGUUGAAGGUGUAU	2	2	0	0	0	0	0	0
ath-miR858a	UUUCGUUGUCUGUUCGACCUU	0	0	0	0	104	98	32	62
ath-miR858b	UUCGUUGUCUGUUCGACCUUG	0	0	0	0	64	64	20	29
ath-miR859	UCUCUCUGUUGUGAAGUCAAA	0	0	0	0	7	3	0	0
ath-miR860	UCAAUAGAUUGGACUAUGUAU	5	4	0	11	11	13	4	6
ath-miR861-3p	GAUGGAUAUGUCUUCAAGGAC	7	8	7	8	3	3	4	6
ath-miR861-5p	CCUUGGAGAAAUAUGCGUCAA	1	0	0	0	0	0	0	0
ath-miR862-3p	AUAUGCUGGAUCUACUUGAAG	0	0	0	0	0	0	0	0
ath-miR862-5p	UCCAAUAGGUCGAGCAUGUGC	1	1	0	0	0	0	0	0
ath-miR863-3p	UUGAGAGCAACAAGACAUAAU	197	160	157	62	2	3	1	1
ath-miR863-5p	UUAUGUCUUGUUGAUCUCAAU	1	0	0	0	0	0	0	0
ath-miR864-3p	UAAAGUCAAUAAUACCUUGAAG	0	0	0	0	1	0	0	0
ath-miR864-5p	UCAGGUAUGAUUGACUUCAAA	24	16	30	0	0	0	0	0
ath-miR865-3p	UUUUUCCUCAAAUUUAUCCAA	0	0	0	0	0	0	0	0
ath-miR865-5p	AUGAAUUUGGAUCUAAUUGAG	0	0	0	0	1	0	0	0
ath-miR866-3p	ACAAAAUCCGUCUUUGAAGA	2	3	3	9	4	5	9	9
ath-miR866-5p	UCAAGGAACGGAUUUUGUUAA	1	2	0	7	0	0	0	0
ath-miR867	UUGAACAUGGUUUAUUAGGAA	0	0	0	0	0	0	0	0
ath-miR868-3p	CUUCUUAAGUGCUGAUAAUGC	0	0	0	0	0	0	0	0
ath-miR868-5p	UCAUGUCGUAAUAGUAGUCAC	0	0	0	0	0	0	0	0
ath-miR869.1	AUUGGUUCAAUUCUGGUGUUG	0	0	0	0	0	0	0	0
ath-miR869.2	UCUGGUGUUGAGAUAGUUGAC	10	12	7	0	107	113	12	3
ath-miR870	UAAUUUGGUGUUUCUUCGAUC	1	0	0	0	0	0	0	0
	**Sum of miRNA Abundance:**	**1,076,444**	**1,400,822**	**864,391**	**816,957**	**123,689**	**108,373**	**50,384**	**48,219**

Leaf

A

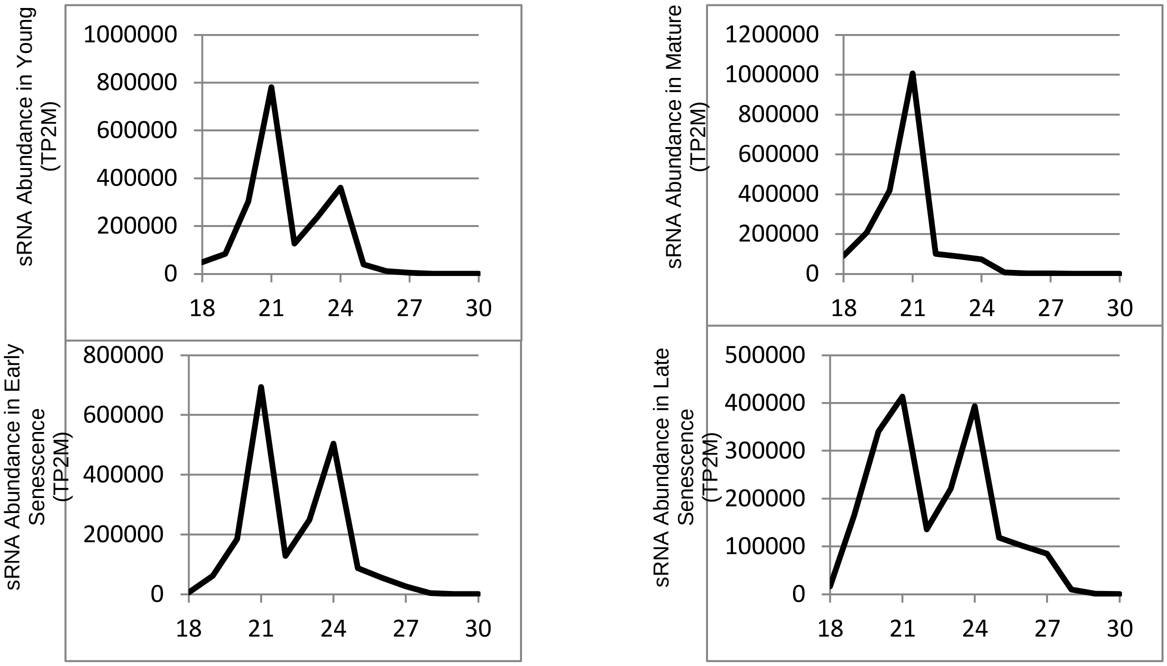

Silique

B

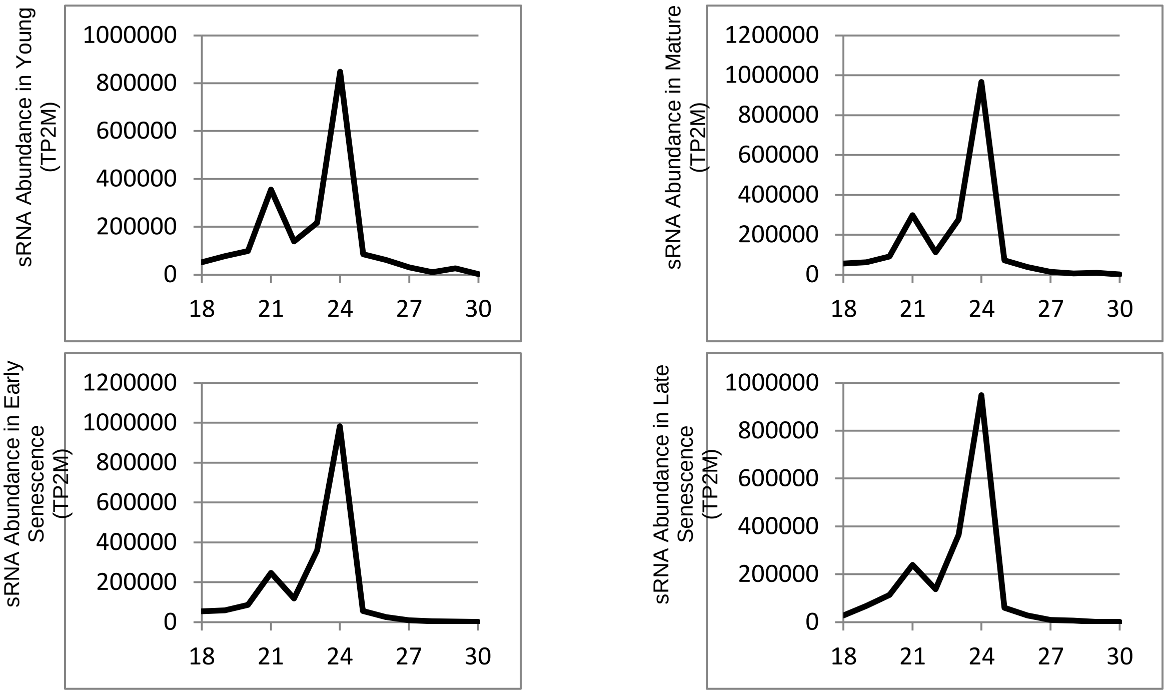

Figure S1. Small RNA size distribution plots.
Genome-matched small RNA data were normalized to transcripts per two million (TP2M) and the total abundance of each size class of small RNA was plotted. (A) Size distributions in young, mature, early senescing and late senescing leaf. (B) Size distributions in young, mature, early senescing and late senescing silique.

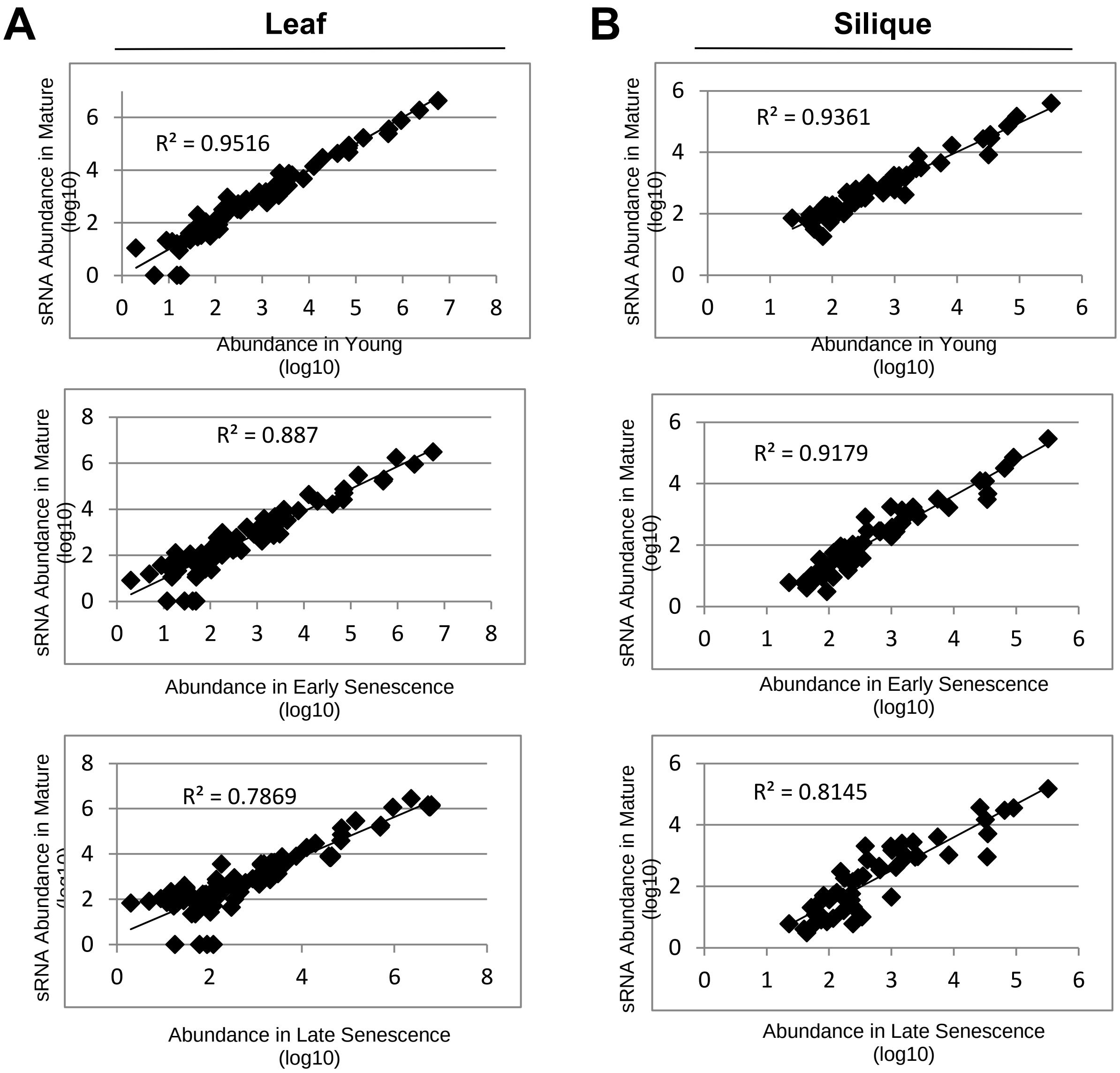

Figure S2. Comparison of miRNA abundance between mature and other stages
miRNA abundance comparison between mature and other stages. Correlation coefficients for each comparison are also shown. (A) Mature leaves compared to young leaves, early senescing leaves and late senescing leaves. (B) Mature siliques compared to young siliques, early senescing siliques and late senescing siliques.
